# Risk factors for residual acetabular dysplasia after closed reduction treatment of developmental dysplasia of the hip: a systematic review and meta-analysis

**DOI:** 10.3389/fped.2025.1694332

**Published:** 2026-01-20

**Authors:** Min Chen, Jun Qian, Li Weng, Ai-Xia Zhang, Ru-Yi Cai

**Affiliations:** Department of the Child Health Department, Women’s Hospital of Nanjing Medical University, (Nanjing Women and Children’s Healthcare Hospital), Nanjing, Jiangsu, China

**Keywords:** closed reduction, developmental dysplasia of the hip (DDH), meta-analysis, residual dysplasia, systematic review

## Abstract

**Background:**

Residual acetabular dysplasia (RAD) is a common complication following closed reduction (CR) for developmental dysplasia of the hip (DDH). This study aims to perform a meta-analysis to identify predictive factors for RAD in order to provide a theoretical basis for early clinical identification and prevention.

**Methods:**

A comprehensive literature search was conducted in PubMed, Embase, Web of Science, and the Cochrane Library databases, covering the period from database inception to November 2024. The quality of the included studies was assessed using the Newcastle–Ottawa Scale, and data analysis was performed using StataSE-64 and RevMan 5.4 software. The odds ratio (OR) and 95% confidence interval (CI) were used for data synthesis. Evidence for all outcomes was graded according to the GRADE system.

**Results:**

This meta-analysis included 16 studies, including a total of 1,338 children who underwent CR for DDH. The analysis identified female sex (OR: 1.96; 95% CI: 1.01–3.81; *p* = 0.05) and femoral head coverage (FHC) (OR: 0.95; 95% CI: 0.92–0.97; *p* = 0.0002) as risk factors for RAD after CR. However, acetabular index (AI) (OR: 1.11; 95% CI: 0.94–1.31; *p* = 0.21), treatment age (<1 year vs. ≥1 year) (OR: 1.16; 95% CI: 0.95–1.42; *p* = 0.13), side of DDH occurrence (OR: 0.84; 95% CI: 0.52–1.36; *p* = 0.48), and number of affected sides (OR: 0.76; 95% CI: 0.05–12.72; *p* = 0.85) were not identified as risk factors for RAD. According to the GRADE assessment, all indicators were rated as “very low-quality evidence,” except for FHC, which was classified as “low-quality evidence.”

**Conclusion:**

The results of this study indicate that female sex and FHC are the primary risk factors for RAD after CR treatment of DDH. Given the inherent limitations of this study, further multicenter prospective clinical studies are needed to clarify the factors contributing to RAD after CR in children with DDH and to implement preventive measures to improve the long-term prognosis of these children.

**Systematic Review Registration:**

PROSPERO CRD420251016618.

## 1 Introduction

Developmental dysplasia of the hip (DDH) is one of the most common structural deformities in children and encompasses a spectrum of abnormalities ranging from mild hip joint underdevelopment to complete hip dislocation (1). The condition occurs more frequently in females, with the left hip more commonly affected than the right (2, 3). DDH is the most common developmental disorder of the lower limbs in pediatric orthopedics and is a major cause of disability in children. Studies have indicated (4, 5) that up to 76% of hip osteoarthritis cases in adults are secondary to untreated DDH in childhood. Without timely diagnosis and intervention, DDH can lead to progressive degenerative changes in the hip joint, ultimately resulting in disability and severely affecting long-term quality of life.

Early diagnosis and treatment of DDH enable concentric reduction of the acetabulum and femoral head at an early stage, allowing the hip joint to develop to a level comparable to that of a normal hip (6, 7). For children aged 6–18 months, closed reduction (CR) under anesthesia followed by spica cast fixation is considered the gold standard for treatment (8, 9). It can successfully treat children who have failed Pavlik harness therapy as well as those with late-diagnosed DDH. However, complications such as iatrogenic avascular necrosis (AVN), redislocation, and residual acetabular dysplasia (RAD) following CR have also raised significant clinical concerns. Among these, RAD remains the most common complication after CR, affecting more than one-third of patients, with its incidence increasing with age. In a study by Huang et al. (10), the rate of incidence of RAD reached 73.6%, the highest reported to date. RAD is often asymptomatic in its early stages, making early detection challenging. If left untreated, it can lead to reduced weight-bearing capacity of the lower limb joints, gait abnormalities, and chronic osteoarthritis, potentially resulting in redislocation and the need for pelvic osteotomy (11, 12). Moreover, certain complications remain inevitable even after osteotomy (13).

Currently, the risk factors for RAD after CR have been extensively studied. Wong et al. (14) conducted a study with an average follow-up of 20 years and identified that the acetabular index and age were important predictive factors for RAD. However, in a study by Dai et al. (15), although the incidence of RAD was higher in children over 18 months and those aged 12–18 months, the age-related difference was not statistically significant. To date, there remains no consensus regarding which factors increase the risk of RAD after CR in children with DDH. This study employed the PICOS framework to define the inclusion and exclusion criteria. We then conducted a systematic review and meta-analysis to identify the risk factors associated with RAD following CR. By synthesizing current clinical evidence and evaluating all relevant variables, this study provides a robust and up-to-date foundation for evidence-based practice. This comprehensive analysis, by pooling data across studies, provides a larger aggregate sample size than individual studies, thereby enhancing statistical power and offering a more robust foundation for evidence-based practice compared with conclusions drawn from single, small-sample studies.

## 2 Materials and methods

### 2.1 Protocol and registration

This systematic review and meta-analysis was conducted in accordance with the PRISMA 2020 statement and was registered in the PROSPERO database (CRD420251016618).

### 2.2 Literature search

The literature search was conducted using PubMed, Web of Science, Embase, and the Cochrane Library. The following terms were used in the search: “residual dysplasia,” “closed reduction,” and “developmental dysplasia of the hip.” The PubMed search query was as follows: [(residual dysplasia) AND (closed reduction)] AND [“Developmental Dysplasia of the Hip"(Mesh) OR Developmental Hip Dysplasia OR Developmental Hip Dislocation OR DDH”]. The search period covered all publications from database inception to November 2024. The search was conducted using a combination of Medical Subject Headings (MeSH) terms and free-text keywords, while also reviewing the references of included studies. EndNote X9 was used to manage and screen all the collected studies. The detailed literature search strategy is depicted in Supplementary Table S1.

### 2.3 Inclusion and exclusion criteria for literature

This study establishes strict inclusion and exclusion criteria based on the PICOS (Population, Intervention, Comparison, Outcome, Study design) framework to ensure a systematic and transparent approach to the clinical question. The inclusion criteria are as follows: (1) The study subjects are DDH patients who have undergone CR; (2) the study reports predictive factors/risk factors for RAD after CR and provides OR values and 95% CI, or OR values and 95% CI can be obtained through data conversion; (3) the study contains clear diagnostic criteria for RAD; and (4) the study is a cohort or case-control study. Exclusion criteria: (1) articles published more than once or based on the same sample; (2) studies with no data or incomplete data; (3) articles unrelated to this study; (4) conference papers, case reports, reviews, or meta-analyses; and (5) articles not published in English.

### 2.4 Literature screening and data extraction

Two researchers independently screened the literature, extracted data, and cross-checked the studies according to the inclusion and exclusion criteria. Any disagreements during this process were discussed and resolved through negotiation between the two researchers. If the disagreement persisted, a senior researcher was consulted to make the final decision. The extracted data included the following: author, publication year, study year, study type, characteristics of the study subjects, predictive factors, diagnostic criteria for RAD, the number of cases by sex (male/female), follow-up duration, treatment age at CR (categorized as <1 year or ≥1 year), and other relevant risk factors.

### 2.5 Literature quality assessment

The quality of the cohort studies and case-control studies was evaluated using the Newcastle–Ottawa Scale (NOS) (16). The NOS evaluates studies across three domains: selection of study groups, comparability between groups, and assessment of exposure or outcome. The maximum score is 9 points, with scores of 0–5 indicating low-to-moderate quality and scores ≥6 indicating high quality. Only high-quality studies were included in this review. The quality assessment was performed independently by two researchers, and any disagreements were resolved through discussion. If consensus could not be reached, a third senior researcher was consulted to make the final decision.

### 2.6 Statistical methods

Statistical analyses were conducted using RevMan 5.4 and StataSE-64 software. The odds ratio (OR) with a 95% confidence interval (CI) was used as the effect measures, and a random-effects model was applied to calculate pooled estimates. A sensitivity analysis was conducted using the leave-one-out method to evaluate the reliability and stability of the results. Publication bias was assessed using funnel plots and Egger's test, with a *p*-value < 0.05 regarded as the threshold for statistical significance. The GRADE framework was applied to assess the quality of evidence for each outcome and classify it as “high,” “moderate,” “low,” or “very low” (17).

## 3 Results

### 3.1 Literature search results

**Figure 1:**
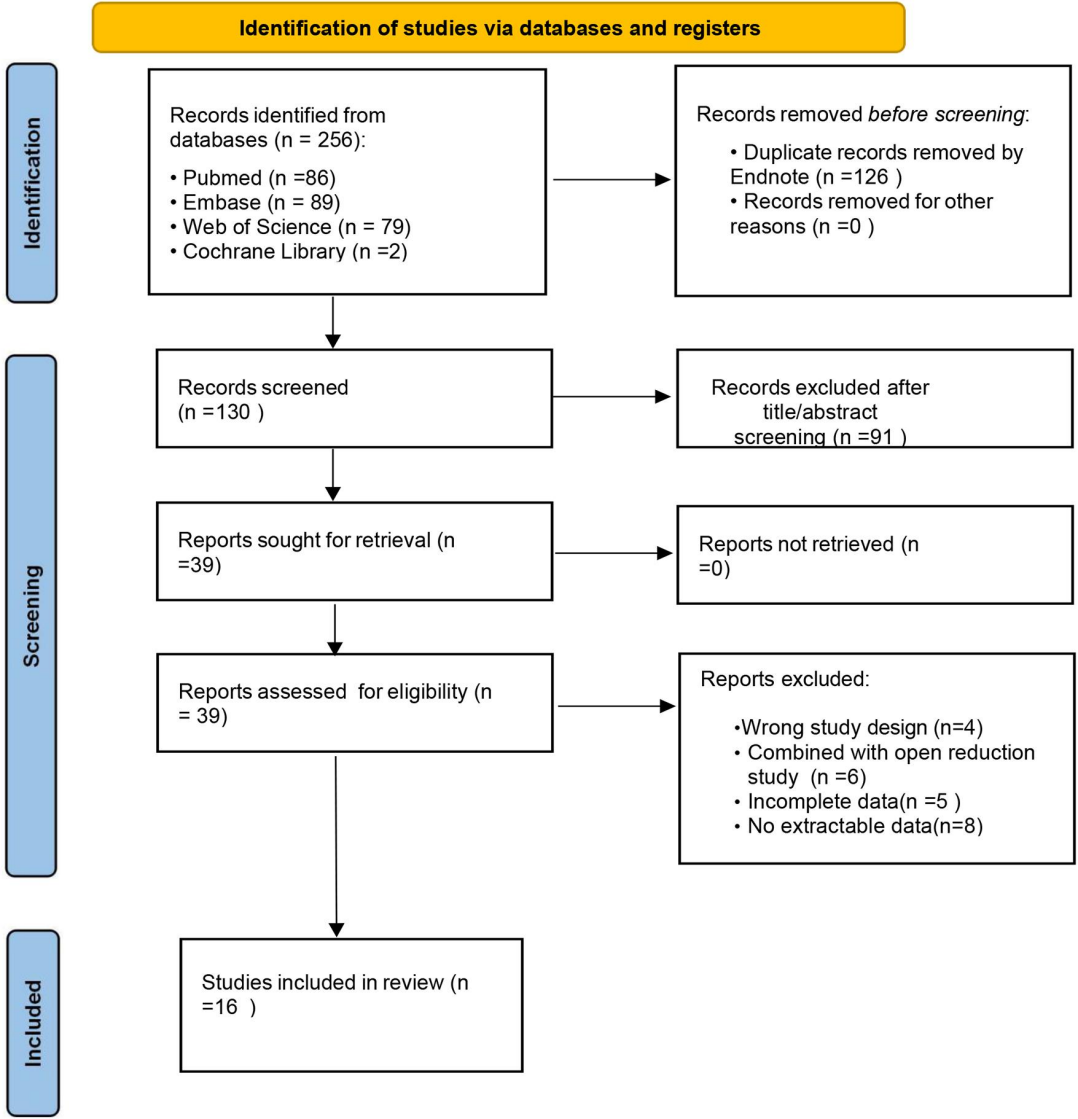
A PRISMA flowchart for the systematic review and meta-analysis.

A total of 256 records were retrieved from the databases. After removing 126 duplicates, 91 records were eliminated by reviewing the titles and abstracts. Based on the inclusion and exclusion criteria, the full texts were then thoroughly examined, and studies that were unrelated to the research topic, lacked data, or were not published in English were excluded. Finally, 16 studies were included in the analysis. Among these, 13 were retrospective, and three were prospective, covering a total of 1,338 children who underwent closed reduction treatment for DDH. A flowchart of literature screening is presented in Figure 1, and the basic characteristics of the included studies are presented in Table 1.

**Table 1:** Basic characteristics of the included articles.

Author	Study period	Region	Study design	Population	Predictive factors	Diagnostic criteria for residual dysplasia	Total hips analyzed (*n*)	Notes (demographics)	Number of cases		Follow-up	Mean/median age^a^	Types of residual dysplasia
									Male	Female			
William 2022	1980–2016	Britain	Retrospective	165 patients with DDH who underwent closed reduction.	Folded limbus and severity of dislocation	X-ray	182	(Case composition not specified in the original study)	NR	NR	9 ± 4.9y	9.8 ± 4.5m	Residual dysplasia, ischemic necrosis
Johnson 2022	2009–2019	USA	Reprospective	48 patients who underwent open or closed reduction.	Gender, age at reduction, CAI, and posterior cartilage acetabular angle	MRI	63	(Case composition not specified in the original study)	NR	NR	35.3 ± 4.9m	9.3 ± 3.2m	Residual dysplasia
Hiroshi 2006	1964–1988	Japan	Retrospective	40 DDH patients who underwent closed reduction with overhead traction (OHT).	Age, severity of dislocation, AI, CE, and CHDD	X-ray	45	[40 patients: 37 females and 3 males; 35 unilateral cases (25 left and 10 right) and 5 bilateral cases]	3 cases	37 cases	15.1–29.5y	9.3m	Acetabular dysplasia
Li 2017	2004–2013	China	Retrospective	89 DDH patients who underwent closed reduction.	AI, CEA, RI, and CHDD	X-ray	99	(89 patients: 99 hips, unilateral 79 patients and bilateral 10 patients)	13 cases	76 cases	61.6 ± 17.7m	16.1 ± 4.6m	Residual acetabular dysplasia
Sankar 2019	2010–2014	USA	Prospective	78 DDH patients who underwent closed reduction.	Age at reduction, previous orthotic treatment, and history of femoral head reducibility	X-ray	87	(78 patients: majority female; median age at CR: 8 months; 33% bilateral cases; 16% breech history; 29% family history of DDH)	10 cases	68 cases	22m	12–36m	Residual dysplasia
Tan 2024	2014–2020	China	Retrospective	DDH children who underwent closed reduction and spica casting for more than 6 months.	FHC, MDP, labral inversion, and quality of reduction	X-ray	110	(102 patients: 94 females and 8 males; 60 left hips, 34 right hips, and 8 bilateral cases; mean age at CR: 14.6 months)	8cases	94cases	58.5 ± 24.8m	14.6 ± 4.7m	Residual acetabular dysplasia/AVN
Zhang 2016	2006–2013	China	Retrospective	126 DDH patients who underwent closed reduction under arthrography guidance.	Arthrography type, FHC, AI, and CE	X-ray	139	(126 patients: 103 females, 23 males; 88 left hips and 51 right hips; mean age at CR: 14 months)	23 cases	103 cases	36m	14m	Residual dysplasia
Meng 2021	2012–2018	China	Prospective	MRI and X-ray evaluation before and after closed reduction in unilateral DDH patients.	LCC	MRI、X-ray	63	(63 patients: 61 females and 2 males; 39 left hips, 24 right hips; mean age at CR: 15.6 months)	2 cases	61 cases	138.6 ± 24.1d	15.6 ± 4.4	Residual acetabular dysplasia
Fu 2023	2016–2020	China	Retrospective	86 DDH patients who underwent closed reduction and spica casting.	LACC and FTD	MRI	92	(86 patients: 81 females and 5 males; 80 unilateral and 6 bilateral; mean age at CR: 13.6 months)	5 cases	81 cases	≥3y	13.6m	Residual dysplasia
Dai 2023	2011–2017	China	Reprospective	82 patients older than 12 months who successfully underwent closed reduction and were followed up for at least 2 years.	AI, AWh, CEA, and FHC	MRI、X-ray	107	(82 patients: 69 females and 13 males; 57 unilateral cases: 33 left and 24 right, and 25 bilateral cases)	13 cases	69 cases	47.8 ± 16.6m	3.8m	Residual dysplasia
Wong 2024	1970–2010	China	Reprospective	DDH patients who underwent closed reduction and were followed up until skeletal maturity.	AI, age, and lateral center-edge angle	X-ray	107	(96 females, 11 males; 62 left hips and 45 right hips; mean age at CR: 8 months; follow-up to skeletal maturity)	11 cases	96 cases	20y	8m	Residual acetabular dysplasia
Arenas-Díaz 2024	2015–2016	Mexico	Reprospective	66 DDH patients who underwent closed reduction with two different fixation techniques.	Modified Lange's “second position”	X-ray	84	[66 patients (gender distribution: 89.3% female and 10.7% male); median age 8 months]	7 cases	59 cases	≥48m	8m	Residual dysplasia, AVN, and redislocation
Ge 2016	2010–2013	China	Prospective	28 DDH patients who underwent closed reduction.	Hip abduction angle, initial AI, final AI, preoperative Tönnis classification, and postreduction FAD	MRI	41	[28 patients (4 males and 24 females); mean age 8.6 months; left 21 hips and right 20 hips]	4 cases	24 cases	17.7m	8.6m	Residual acetabular dysplasia/AVN
Yasin 2022	2016–2019	Jordan	Reprospective	47 patients who underwent closed reduction with long or short spica casting.	Long spica casting and short spica casting	CT	47	[47 patients (91.5% female, 8.5% male); median age 7.83 months; 19 bilateral cases (40.4%)]	4 cases	43 cases	1y	7.83m	Residual dysplasia, AVN, redislocation
Huang 2022	2011–2017	China	Reprospective	104 DDH patients who underwent closed reduction and were followed up for at least 2 years.	TSL, AI, age, and IHDI classification	MRI	125	[104 patients (13 males and 91 females); mean age 18.3 months]	13 cases	91 cases	50.2m	18.3m	Residual acetabular dysplasia
Zhang 2020	2011–2013	China	Reprospective	107 DDH patients who underwent closed reduction.	Age	X-ray	156	[107 patients (12 males and 95 females); median age 13.0 months]	12 cases	95 cases	6.7 ± 0.8y	13.0 ± 5.4m	Residual dysplasia, AVN, and redislocation

^a^ Age at closed reduction, presented as mean/median. For meta-analysis, categorized as <1 year vs. ≥1 year.

### 3.2 Literature quality assessment

The 16 included studies (10, 14, 15, 18–30) were evaluated for methodological quality using the NOS, with scores ranging from 6 to 9, indicating that the overall quality of the included literature was high. The detailed quality assessment results can be found in Supplementary Tables S2, S3.

### 3.3 Meta-analysis

#### 3.3.1 Treatment age (<1 year vs. ≥1 year)

**Figure 2:**
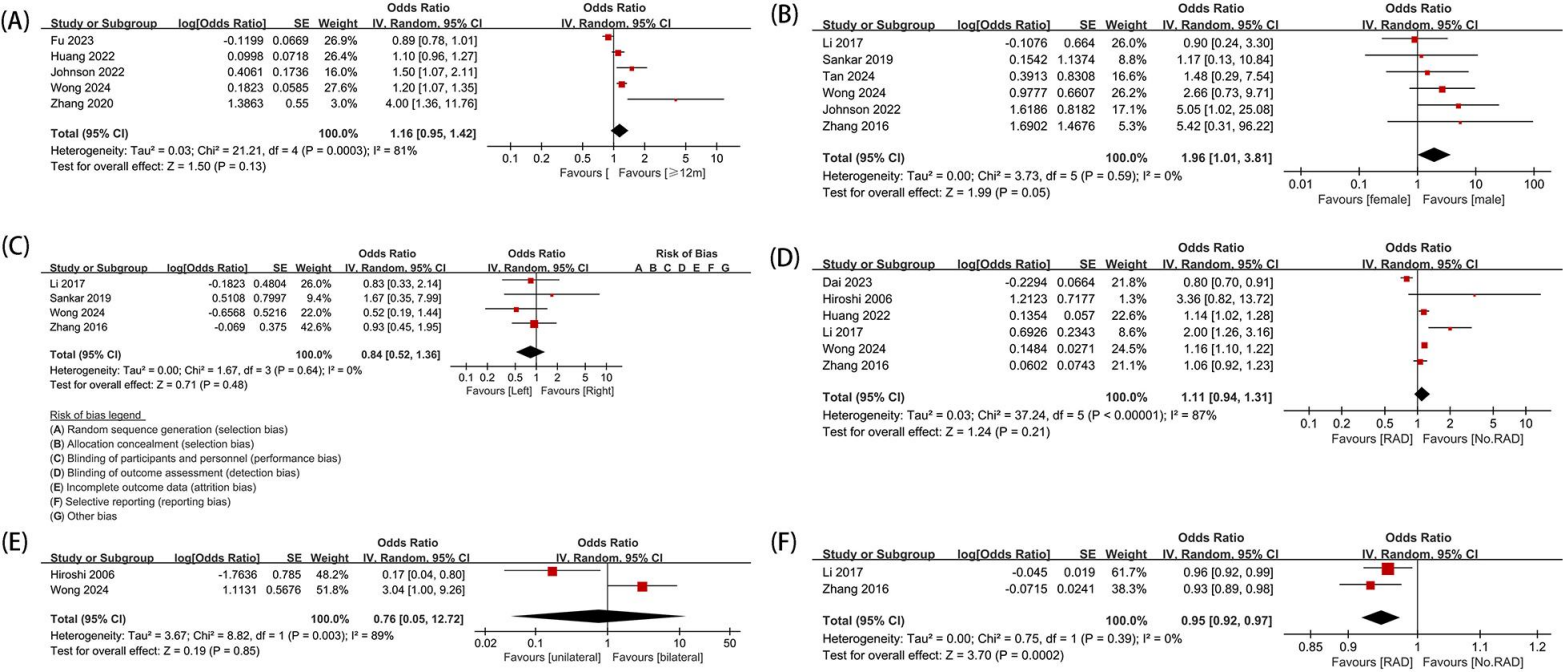
Forest map. **(A)**Treatment age; **(B)** gender; **(C)** side (left/right); **(D)** acetabular index (AI); **(E)** number of affected sides in DDH (unilateral/bilateral); and **(F)** femoral head coverage (FHC).

A total of five studies were included. The results showed no statistically significant difference between children aged <1 year and those aged ≥1 year (OR: 1.16; 95% CI: 0.95–1.42); however, the substantial heterogeneity (*I*^2^ = 81%) precludes a definitive conclusion regarding the association between treatment age (dichotomized at 1 year) and RAD (Figure 2A).

#### 3.3.2 Gender

A total of six studies were included. The results showed a significant difference in the occurrence of RAD after CR between females and males (OR: 1.96; 95% CI: 1.01–3.81; *I*^2^ = 0%), indicating that being female is a risk factor for RAD, with a significantly higher incidence in females compared with males (Figure 2B).

#### 3.3.3 Side (left/right)

A total of four studies were included. The statistical analysis showed no significant difference in the occurrence of RAD between the left and the right hips (OR: 0.84; 95% CI: 0.52–1.36; *I*^2^ = 0%), suggesting that the side of the affected hip is not a risk factor for RAD after CR treatment (Figure 2C).

#### 3.3.4 Acetabular index

A total of six studies were included. The results did not show a significant association between the acetabular index (AI) after CR and the occurrence of RAD (OR: 1.11; 95% CI: 0.94–1.31; *I*^2^ = 87%), indicating that AI is not a risk factor for this complication (Figure 2D).

#### 3.3.5 Number of affected sides in DDH (unilateral/bilateral)

A total of two studies were included. The results of the analysis showed no significant difference in the occurrence of RAD after CR treatment, regardless of whether the hip joint had unilateral or bilateral DDH (OR: 0.76; 95% CI: 0.05–12.72; *I*^2^ = 89%) (Figure 2E).

#### 3.3.6 Femoral head coverage

A total of 2 studies were included. The results showed a significant association between femoral head coverage (FHC) and the occurrence of RAD after CR (OR: 0.95; 95% CI: 0.92–0.97; *I*^2^ = 0%). These findings indicate that FHC can serve as a predictive factor for RAD after closed reduction treatment (Figure 2F).

#### 3.3.7 Other indicators

In addition to the above indicators, which were analyzed based on two studies, there are 18 other indicators, each examined in only one study. Because of the limited data, these indicators were not included in the meta-analysis. A systematic summary of these factors is presented below.

The Wiberg center-edge angle (CEA) (21) (OR = 0.0744, 95% CI: 0.0208–0.2666, *p* = 0.0001), preoperative AI (15) (OR = 1.225, 95% CI: 1.025–1.464, *p* = 0.026), cartilage acetabular index (CAI) > 23 (26) (OR = 1.133, 95% CI: 1.006–1.276, *p* = 0.040), posterior cartilage acetabular angle (26) (OR = 0.763, 95% CI: 0.599–0.972, *p* = 0.029), Reimers’ index (RI) (21) (OR = 1.086, 95% CI: 1.017–1.161, *p* = 0.014), femoral head to triradiate cartilage distance (FTD) (26) (OR = 0.514, 95% CI: 0.307–0.859, *p* = 0.011), immobilization duration (26) (OR = 1.054, 95% CI: 1.006–1.104, *p* = 0.027), preoperative acetabular width (AWh) (15) (OR = 0.006, 95% CI: 0.01–0.384, *p* = 0.016), AWh (15) (OR = 0.026, 95% CI: 0.002–0.296, *p* = 0.0053), Tönnis classification grade III/IV (14) (OR = 4.80, 95% CI: 1.147–20.085, *p* = 0.032), and teardrop line (TSL) (10) (OR = 0.214, 95% CI: 0.083–0.552, *p* = 0.001) were identified as risk factors for RAD.

On the other hand, family history (22) (OR = 3.333, 95% CI:0.689–16.126, *p* = 0.134), femoral head width (26) (OR = 1.253, 95% CI:0.97–1.617, *p* = 0.0849), center-head distance difference (CHDD) (21) (OR = 0.956, 95% CI:0.814–1.123, *p* = 0.583), femoral head ossification nucleus (23) (OR = 0.446, 95% CI:0.174–1.148, *p* = 0.093), limbus inversion (23) (OR = 0.262, 95% CI:0.018–3.814, *p* = 0.327), and limbus folding (18) (OR = 1.285, 95% CI:0.599–2.756, *p* = 0.520) were not considered risk factors for RAD (Table 2).

**Table 2:** Analysis of other indicators for RAD.

Outcome	OR	95% CI	*p*
Wiberg center-edge angle (CEA)	0.0744	0.0208–0.2666	0.0001
Preoperative acetabular index (AI)	1.225	1.025–1.464	0.026
Cartilage acetabular index (CAI)>23	1.133	1.006–1.276	0.040
Posterior cartilage acetabular angle	0.763	0.599–0.972	0.029
Reimers’ index (RI)	1.086	1.017–1.161	0.014
Femoral head to triradiate cartilage distance (FTD)	0.514	0.307–0.859	0.011
Immobilization duration	1.054	1.006–1.104	0.027
Preoperative acetabular width (AWh)	0.006	0.01–0.384	0.016
Acetabular width (AWh)	0.026	0.002–0.296	0.0053
Tönnis classification grade III/IV	4.80	1.147–20.085	0.032
Teardrop and teardrop line (TSL)	0.214	0.083–0.552	0.001
Family history	3.333	0.689–16.126	0.134
Femoral head width	1.253	0.97–1.617	0.0849
Center-head distance difference (CHDD)	0.956	0.814–1.123	0.583
Femoral head ossification nucleus	0.446	0.174–1.148	0.093
Limbus inversion	0.262	0.018–3.814	0.327
Limbus folding	1.285	0.599–2.756	0.520
Complete concentric reduction	5.4167	1.249–23.489	0.002
AI <30° and CEA >5° at the age of 5 or 6 years	4.889	1.246–19.190	0.023

CEA, center-edge angle; RI, Reimers index; FTD, femoral head to triradiate cartilage distance; CAI, cartilage acetabular index; AWh, acetabular width; CHDD, center-head distance difference.

Complete concentric reduction (28) (OR = 5.4167, 95% CI: 1.249–23.489, *p* = 0.002) and AI <30° and CEA >5° at the age of 5 or 6 years (20) (OR = 4.889, 95% CI: 1.246–19.190, *p* = 0.023) were identified as protective factors against RAD (Table 2).

### 3.4 Sensitivity analysis

**Figure 3:**
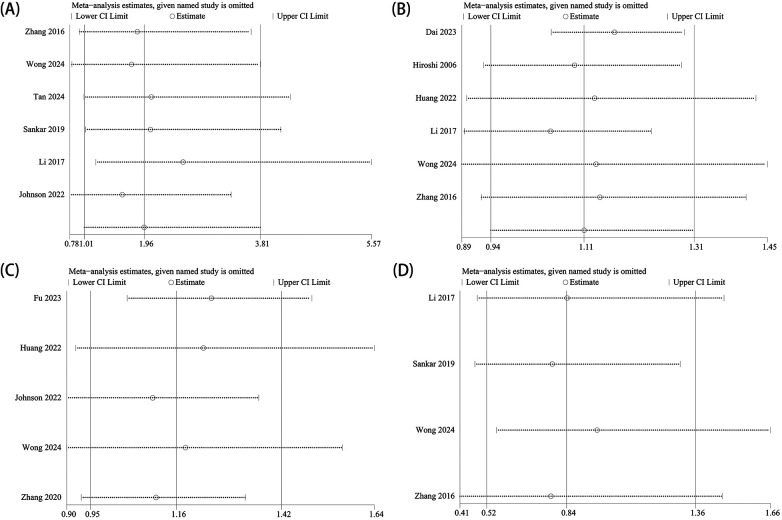
Sensitivity analysis. **(A)** Gender; **(B)** acetabular index (AI); **(C)** treatment age; and **(D)** side (left/right).

A leave-one-out approach was applied for the sensitivity analysis to evaluate the risk factors for RAD after CR in children with DDH. The results showed that after excluding the studies by Zhang et al. (24), Wong et al. (14), and Johnson et al. (19), the significance of the sex indicator changed from significant to non-significant (Figure 3A). After excluding the studies by Hiroshi et al. (20), Huang et al. (10), Li et al. (21), Wong et al. (14), and Zhang et al. (24), the significance of the AI indicator changed from non-significant to significant (Figure 3B). After excluding the studies by Huang et al. (10), Johnson et al. (19), Wong et al. (14), and Zhang et al. (30), the significance of the treatment age indicator in the combined subgroup changed from non-significant to significant (Figure 3C). Excluding any individual study did not affect the significance of the left/right side combined subgroup (Figure 3D).

### 3.5 Publication bias

**Figure 4:**
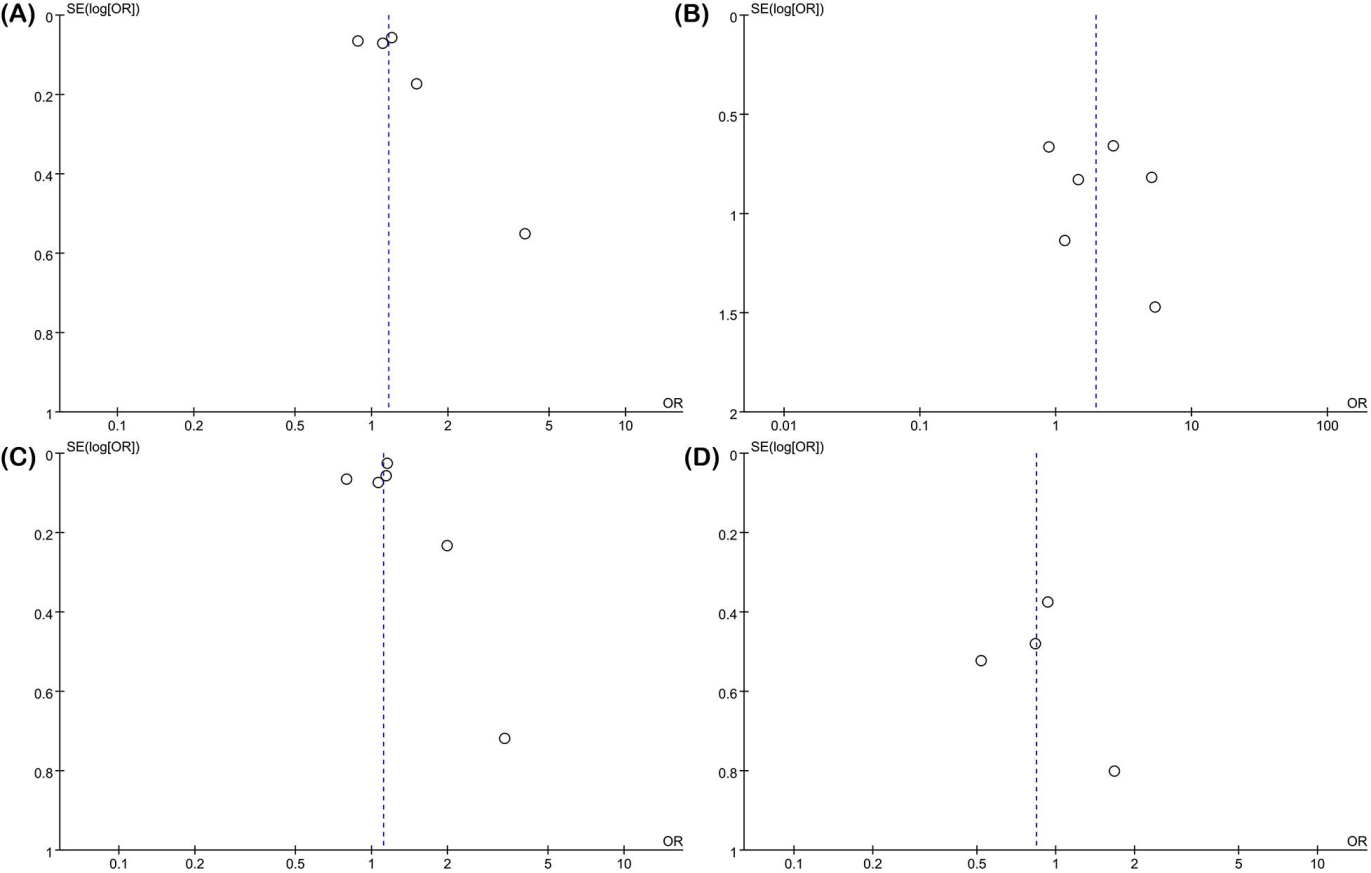
Funnel map. **(A)** Treatment age; **(B)** gender; **(C)** acetabular index (AI); and **(D)** side (left/right).

Egger's test and funnel plots were used to assess publication bias. The results of Egger's test indicated no significant publication bias for treatment age (*P* = 0.281), sex (*P* = 0.589), AI (*P* = 0.892), and side (*P* = 0.702). Similarly, the funnel plots confirmed the absence of publication bias for treatment age (Figure 4A), sex (Figure 4B), AI (Figure 4C), and side (Figure 4D). For the remaining indicators, due to the limited number of studies, publication bias analysis could not be performed.

### 3.6 GRADE rating

In the GRADE assessment of indicators such as sex, side (left/right), number of affected hip joints (unilateral/bilateral), AI, FHC, and treatment age, the FHC indicator was classified as “low.” Meanwhile, the indicators sex, side, number of affected hip joints, AI, and treatment age were classified as “very low” quality. Detailed GRADE analysis results can be found in Table 3.

**Table 3:** GRADE rating of outcomes.

Risk factors	No. of studies	OR	95% CI	*I*^2^; *P*-value	Risk of bias	Inconsistency	Indirectness	Imprecision	Publication bias	Plausible confounding	Magnitude of effect	Dose–response gradient	GRADE
Gender (female vs. male)	6	1.96	1.01, 3.81	0%; *P* = 0.59	No serious risk	No serious inconsistency	No serious indirectness	Serious imprecision	Undetected	Would not reduce effect	No	No	Very low
Side (left vs. right)	4	0.84	0.52, 1.36	0%; *P* = 0.64	No serious risk	No serious inconsistency	No serious indirectness	Serious imprecision	Undetected	Would not reduce effect	No	No	Very low
DDH involvement (unilateral vs. bilateral)	2	0.76	0.05, 12.72	89%; *P* = 0.003	No serious risk	Serious inconsistency	No serious indirectness	Serious imprecision	NA	Would not reduce effect	No	No	Very low
AI	6	1.11	0.94, 1.31	87%; *P*<0.001	No serious risk	Serious inconsistency	No serious indirectness	Serious imprecision	Undetected	Would not reduce effect	No	No	Very low
FHC	2	0.95	0.92, 0.97	0%; *P* = 0.39	No serious risk	No serious inconsistency	No serious indirectness	No serious imprecision	NA	Would not reduce effect	No	No	Low
Treatment age (<1 year vs. ≥1 year)	5	1.16	0.95, 1.42	81%; *P* = 0.0003	No serious risk	Serious inconsistency	No serious indirectness	Serious imprecision	Undetected	Would not reduce effect	No	No	Very low

## 4 Discussion

RAD is the most common complication after CR for DDH. Multiple long-term follow-up studies have found that the rate of RAD after CR is as high as 35%–58% (11, 31, 32). In addition, the rate of prevalence of secondary osteoarthritis caused by acetabular dysplasia after DDH treatment is reported to range between 43% and 50% (33, 34). Therefore, early identification of hips that do not demonstrate adequate improvement and are at risk for RAD after CR is crucial for guiding subsequent management.

This study identified several potential risk factors for RAD after CR, including female sex, FHC, CEA, preoperative AI, CAI > 23, cartilage acetabular posterior angle, RI, distance from the femoral head to the triradiate cartilage, duration of immobilization, AWh, AWh, and Tönnis classification III/IV, as well as the teardrop and TSL. Conversely, complete concentric reduction and an AI <30° or CEA >5° at 5 or 6 years of age were identified as protective factors against RAD after CR. However, there remains disagreement regarding the relationship between treatment age (at 1-year cutoff) and the occurrence of RAD.

Our findings primarily revealed that female sex and the FHC were the main risk factors for the occurrence of RAD after CR. It also found that factors such as the side of DDH occurrence, the number of affected hip joints, AI, and treatment age did not appear to have a clear association with RAD. In the sensitivity analysis of indicators with more than three studies, the study found that the factor of the side of DDH occurrence was stable, while factors such as sex, AI, and treatment age (as dichotomized) were unstable. For these unstable indicators, further research may be needed to confirm these findings, as instability suggests that the results may vary and be overly influenced by individual studies. Therefore, additional evidence is needed to reinforce these conclusions.

In addition, this analysis confirmed that none of the indicators showed publication bias, suggesting that these findings are relatively reliable. All risk factors were analyzed using the GRADE system, in which FHC was identified as the most reliable indicator, with a “low quality” level. The remaining indicators were all classified as “very low quality.” Therefore, in clinical practice, more attention should be paid to FHC, as it demonstrates a relatively stable and reliable association with RAD.

This study is the first meta-analysis to systematically evaluate the risk factors associated with RAD following CR in children with DDH. The results indicate that female sex is a significant predictor of RAD, extending the understanding of its influence beyond the initial onset of DDH to treatment outcomes. Notably, our findings align with those of a recent meta-analysis by Chen et al., which identified female sex as an important risk factor for the development of DDH (35). Rather than representing a repetition of existing knowledge, our results highlight the consistently critical role of female sex across the spectrum of DDH, right from its occurrence and treatment to long-term prognosis.

Existing literature primarily explains the observed sex differences from two perspectives. First, differences in skeletal anatomy may be a fundamental cause. Studies suggest that, compared with males, the inherent structural characteristics of the female acetabulum may render the hip joint less stable during development. This may predispose females to DDH and may also negatively influence postreduction acetabular remodeling outcomes (36). Second, endocrine factors are considered crucial. Some researchers propose that higher estrogen levels in females may lead to increased ligamentous laxity, thereby further exacerbating hip joint instability (37, 38). This hormone-mediated biomechanical environment may simultaneously influence both the susceptibility to DDH and the potential for posttreatment recovery.

Based on the above findings and discussion, this study carries clear clinical significance. It suggests that in the clinical management of DDH, particularly during post-treatment follow-up, special attention should be paid to female patients. For these children, clinicians should consider implementing closer and longer-term imaging follow-up and monitoring to facilitate the timely detection of RAD signs and enable intervention. This approach helps optimize the allocation of medical resources and ultimately improves the long-term prognosis for female DDH patients.

According to the results, FHC was identified as the most significant risk factor compared with the other variables. FHC refers to the proportion of the femoral head covered by the acetabulum, reflecting both the depth of femoral head insertion and acetabulum stability. One study (23) pointed out that during intraoperative joint imaging, an FHC <30% was associated with persistent developmental dysplasia in more than 80% of hips. FHC may serve as an early warning indicator for RAD, aiding in the identification of high-risk patients. For those with lower FHC values, enhanced postoperative monitoring is warranted. Future studies should investigate whether adjunctive interventions—such as bracing or early osteotomy—can improve outcomes in this high-risk subgroup. Greater clinical attention should be devoted to this indicator. Protective factors such as complete concentric reduction and an AI < 30° or CEA > 5° at 5–6 years of age may serve as useful references for prognostic evaluation. The inconsistent findings regarding other variables such as AI and treatment age suggest the need for individualized assessment and caution against reliance on any single parameter.

This analysis has several limitations. First, the overall sample size is relatively small, and each indicator is supported by only a limited number of studies, which may introduce selection bias or confounding factors. Second, most of the included studies are from Asian countries, leading to greater heterogeneity for some indicators, which could affect the results, and the generalizability of the conclusions requires further validation. Third, the sensitivity analysis revealed that indicators such as sex, AI, and treatment age were unstable, and their evidence level may be reduced due to individual studies. Fourth, in the GRADE assessment, only one indicator was rated as low-quality evidence, while the rest were classified as very low quality. Fifth, this study was unable to perform a meta-analysis on the severity of preoperative dislocation (such as Tönnis or IHDI classification). Sixth, this study was also unable to quantify the impact of intraoperative techniques and surgeon-related factors—such as surgeon experience, specific reduction maneuvers, stability assessment, and postoperative immobilization protocols—on the occurrence of RAD. Finally, this study did not analyze the potential interaction between RAD and another significant complication—AVN.

## 5 Conclusion

This study is the first to rigorously demonstrate, through a meta-analysis, that female sex and FHC are the primary risk factors for RAD following CR in children with DDH, providing an important basis for early clinical identification and risk stratification of high-risk patients. Notably, FHC can serve as an objective intraoperative assessment indicator. Although this study lays a foundation for future research, its conclusions are limited by the small sample size and evidence quality. Consequently, further multicenter prospective clinical studies are warranted to validate these associations and to develop effective preventive strategies for improving long-term patient outcomes.

## Data Availability

The original contributions presented in the study are included in the article/Supplementary Material, and further inquiries can be directed to the corresponding author.
