# Supplementary material for: Risk factors for residual acetabular dysplasia after closed reduction treatment of developmental dysplasia of the hip: a systematic review and meta-analysis
Source: Front Pediatr. 2026 Jan 20;13:1694332. doi: 10.3389/fped.2025.1694332 (PMC12864423; doi:10.3389/fped.2025.1694332)
Supplement: Supplementary file 1 [file Table1.docx]

**Supplementary materials**

Table S1 The search strategy

| Datebases | Searching strategy | Literature number |
| --- | --- | --- |
| Pubmed | (((residual) AND (dysplasia)) AND (closed reduction)) AND (("Developmental Dysplasia of the Hip"[Mesh]) OR (((((((((Developmental Hip Dysplasia) OR (Developmental Hip Dysplasias)) OR (Dysplasia, Developmental Hip)) OR (Hip Dysplasia, Developmental)) OR (Hip Dislocation, Developmental)) OR (Developmental Hip Dislocations)) OR (Dislocation, Developmental Hip)) OR (Developmental Hip Dislocation)) OR (DDH))) | 86 |
| Web of science | (((residual) AND (dysplasia)) AND (closed reduction)) AND ((Developmental Dysplasia of the Hip) OR (((((((((Developmental Hip Dysplasia) OR (Developmental Hip Dysplasias)) OR (Dysplasia, Developmental Hip)) OR (Hip Dysplasia, Developmental)) OR (Hip Dislocation, Developmental)) OR (Developmental Hip Dislocations)) OR (Dislocation, Developmental Hip)) OR (Developmental Hip Dislocation)) OR (DDH))) | 89 |
| Embase | (((residual) AND (dysplasia)) AND (closed reduction)) AND ((Developmental Dysplasia of the Hip) OR (((((((((Developmental Hip Dysplasia) OR (Developmental Hip Dysplasias)) OR (Dysplasia, Developmental Hip)) OR (Hip Dysplasia, Developmental)) OR (Hip Dislocation, Developmental)) OR (Developmental Hip Dislocations)) OR (Dislocation, Developmental Hip)) OR (Developmental Hip Dislocation)) OR (DDH))) | 76 |
| Cochrane | (((residual) AND (dysplasia)) AND (closed reduction)) AND ((Developmental Dysplasia of the Hip) OR (((((((((Developmental Hip Dysplasia) OR (Developmental Hip Dysplasias)) OR (Dysplasia, Developmental Hip)) OR (Hip Dysplasia, Developmental)) OR (Hip Dislocation, Developmental)) OR (Developmental Hip Dislocations)) OR (Dislocation, Developmental Hip)) OR (Developmental Hip Dislocation)) OR (DDH))) | 2 |

Table S2. Quality evaluation of the eligible studies with Newcastle–Ottawa scale.

| Study | Selection | | | | Comparability | | Outcome | | |
| --- | --- | --- | --- | --- | --- | --- | --- | --- | --- |
|  | Representative-ness | Selection of  non-exposed | Ascertainment  of exposure | Outcome not present at start | Comparability on most important factors | Comparability on other risk factors | Assessment of outcome | Long enough follow-up (median≥1 year) | Adequacy  (completeness) of follow-up |
| William 2022 | * | * | * | * | * | - | * | * | * |
| Hiroshi 2006 | * | * | * | * | - | - | * | * | * |
| Li 2017 | * | * | * | * | * | - | * | * | * |
| Sankar 2019 | * | * | * | * | * | * | * | * | * |
| Tan 2024 | * | * | * | * | * | - | * | * | * |
| Zhang 2016 | * | * | * | * | * | - | * | * | * |
| Meng 2021 | * | * | * | * | * | - | * | * | * |
| Fu 2023 | * | * | * | * | * | - | * | * | * |
| Dai 2023 | * | * | * | * | * | - | * | * | * |
| Wong 2024 | * | * | * | * | * | - | * | * | * |
| Arenas-Díaz 2024 | * | * | * | * | * | - | * | * | * |
| Ge 2016 | * | * | * | * | * | - | * | * | * |
| Yasin 2022 | * | * | * | * | * | - | * | * | * |
| Huang 2022 | * | * | * | * | * | - | * | * | * |
| Zhang 2020 | * | * | * | * | * | - | * | * | * |

*indicates criterion met; - indicates significant of criterion not met.

Table S3. Quality evaluation of the eligible studies with Newcastle–Ottawa scale.

| Study | Selection | | | | Comparability | | Outcome | | |
| --- | --- | --- | --- | --- | --- | --- | --- | --- | --- |
|  | Case identification is appropriate | Case representation | Contrast selection | Determination of contrast | Comparability on most important factors | Comparability on other risk factors | exposed factors | Take the same approach | Nonresponse rate |
| Johnson 2022 | * | * | * | * | * | - | * | - | * |

*indicates criterion met; - indicates significant of criterion not met.
